# Qualitätsmanagement und -entwicklung in der Primärprävention und Gesundheitsförderung: Stand, Herausforderungen und Perspektiven

**DOI:** 10.1007/s00103-022-03494-2

**Published:** 2022-02-16

**Authors:** Ulla Walter

**Affiliations:** Institut für Epidemiologie, Sozialmedizin und Gesundheitssystemforschung, Medizinische Hochschule Hannover, 30625 Hannover, Deutschland

**Keywords:** Qualitätsentwicklung, Konzeptqualität, Qualitätsinstrumente, Zertifizierung, Präventionsgesetz, Quality development, Concept quality, Quality instruments, Certification, Prevention Act

## Abstract

In den vergangenen 3 Jahrzehnten nahm die Qualitätsentwicklung in der Primärprävention und Gesundheitsförderung eine deutliche Entwicklung. Wesentlich dazu beigetragen hat ihre gesetzliche Verankerung. Einen Schub gaben die Wiedereinführung der Prävention und Gesundheitsförderung in der gesetzlichen Krankenversicherung und das Präventionsgesetz. Die damit bereits im Vorfeld begonnenen Diskurse unter Einbindung zahlreicher Forschender, Träger und Akteure unterschiedlicher Felder der Prävention und Gesundheitsförderung trugen zu einem vermehrten Verständnis zentraler Aspekte der Qualitätsentwicklung bei. Inzwischen liegen für die Prävention und Gesundheitsförderung umfassende Verfahren zur Qualitätssicherung und -entwicklung vor. Zudem sind für alle 4 Qualitätsdimensionen – Planungs‑, Struktur‑, Prozess- und Ergebnisqualität – Handlungsempfehlungen, Checklisten, Instrumente etc. aufbereitet und leicht zugänglich. Zertifizierungen und Qualitätssiegel für Interventionen und gesundheitsförderliche Einrichtungen sind verfügbar.

Allerdings wird keines der genannten Verfahren flächendeckend und kontinuierlich eingesetzt. Die Bereiche in der Prävention und Gesundheitsförderung unterscheiden sich deutlich hinsichtlich der Umsetzung der Qualitätssicherung. Hindernisse sind u. a. unzureichende personelle und finanzielle Ressourcen bei gleichzeitiger Diskontinuität sowie einrichtungsinterne Qualitätsmanagementsysteme. Handlungsbedarf besteht bei der Weiterentwicklung der Umsetzung von Qualität vor Ort, insbesondere im Setting Kommune, und der Integration der Qualitätssicherung in die bestehenden Strukturen. Qualifizierung, ein intensiver Austausch sowie eine kleinräumige Präventionsberichterstattung sollten die Qualitätsentwicklung fördern.

## Abstract (en)

In the past three decades, quality development in primary prevention and health promotion have developed significantly. A major contribution to this has been their legal anchoring. The reintroduction of prevention and health promotion in the statutory health insurance and the Prevention Act gave this development an additional boost. The discourse that had already begun in the run-up to this, involving numerous researchers and stakeholders from different fields of prevention and health promotion, contributed to an increased understanding of central aspects of quality development. Comprehensive procedures for quality assurance and development are available for prevention and health promotion. In addition, recommendations for action, checklists, and instruments, for example, have been edited and are easily accessible for all four quality dimensions – planning, structural, process, and outcome quality. Certifications and quality seals for interventions and health promoting institutions are available.

However, none of these procedures is used comprehensively and continuously. The fields in prevention and health promotion differ significantly with regard to the implementation of quality assurance. Obstacles are, among other things, insufficient human and financial resources with simultaneous discontinuity and intra-facility quality management systems. There is a need for action to further develop the implementation of quality on site, especially in the community setting, and to better integrate quality assurance into existing structures. Qualification, intensive exchange, and small-scale prevention reporting should promote quality development.

## Einleitung

Der Beitrag gibt einen Überblick über die Qualitätsentwicklung im Sinne der kontinuierlichen Verbesserung [1] in der Primärprävention und Gesundheitsförderung in Deutschland. Dieses Feld zeichnet sich durch Interventionen unterschiedlicher Komplexität aus – von einfachen Maßnahmen über hochstrukturierte verhaltensbezogene Programme bis hin zu Settingansätzen und kommunalen Strukturen. Charakteristisch ist ein breites Spektrum an Akteuren (z. B. Bundeszentrale für gesundheitliche Aufklärung (BZgA), Landesvereinigungen für Gesundheit, Krankenkassen), Einrichtungen (z. B. Betriebe, Kitas, Schulen, Pflegeheime) und Trägern (z. B. öffentliche, private, gemeinnützige, konfessionelle) mit ihren spezifischen (Finanzierungs‑)Strukturen, die in der Regel nicht exklusiv auf Prävention und Gesundheitsförderung ausgerichtet sind. Diese unterliegen vielfach gesetzlich vorgegebenen Anforderungen an Qualitätssicherung und/oder einrichtungsspezifischen Qualitätsmanagementsystemen mit unterschiedlichen Perspektiven (z. B. Gesundheit, Bildung, soziale Arbeit; [2]). Vor diesem Hintergrund ist es nicht verwunderlich, dass in der Prävention und Gesundheitsförderung bis heute unterschiedliche Erfahrungen, Voraussetzungen und Anforderungen bezüglich Qualitätssicherung und Qualitätsentwicklung [3] bestehen.

**Figure 1:**
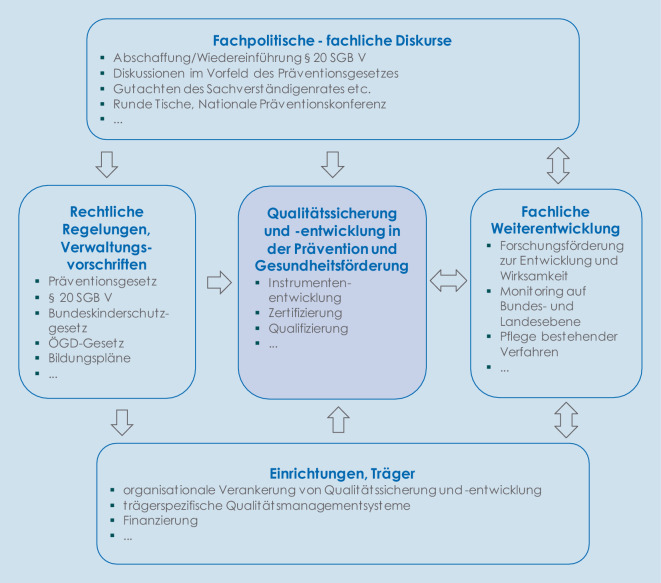

Ziel des Beitrages ist es, einen Einblick in Qualitätssicherung und -management in der Prävention und Gesundheitsförderung zu geben sowie Meilensteine der Qualitätsentwicklung und Herausforderungen aufzuzeigen. Betrachtet werden gesetzliche Regelungen ebenso wie fachliche Entwicklungen und Diskurse. Die Abb. 1 zeigt verschiedene Bereiche, die die Qualitätsentwicklung in der Prävention und Gesundheitsförderung beeinflussen. Diese werden im Beitrag näher beschrieben. Zur Definition von Qualitätssicherung, Qualitätsmanagement und Qualitätsentwicklung siehe [1].

## Entwicklung in den letzten Jahrzehnten

Seit den 1990er Jahren haben Qualitätssicherung und -management eine deutliche Entwicklung genommen und nach anfänglicher Skepsis bis Ablehnung eine breitere Akzeptanz erfahren. Diese ist eng verbunden mit der vermehrten politischen Anerkennung, Förderung und teilweisen gesetzlichen Verankerung von Prävention und Gesundheitsförderung. Inzwischen sind Begriffe weitgehend geklärt, so sind die Sicherung und Verbesserung der Qualität als Aufgabe der Akteure gegenüber der externen wissenschaftlichen Evaluation abgegrenzt. Bezüglich der Anforderungen an die Konzeption und Umsetzung von Prävention und Gesundheitsförderung besteht weitgehend Einigkeit. Entsprechende Empfehlungen, Überblicke über Qualitätsinstrumente und Methoden für unterschiedliche Handlungsfelder und Settings liegen leicht zugänglich und aufbereitet für ein breites Fachpublikum vor (z. B. [4, 5]). Bücher für Praktikerinnen und Praktiker, Studierende und Dozierende fassen zentrale Inhalte zusammen (z. B. [6]). Zu dieser Entwicklung haben in den vergangenen Jahrzehnten zahlreiche Expertisen und Empfehlungen (u. a. [7]), wissenschaftliche Projekte (u. a. [8, 9]) und Diskurse beigetragen. Eine breite und intensive Auseinandersetzung mit der Prävention und Gesundheitsförderung unter Einbezug unterschiedlicher Einrichtungen und Betätigungsfelder erfolgte besonders im Rahmen des in 4 Legislaturperioden diskutierten und 2015 in Kraft getretenen Präventionsgesetzes (PrävG).

Die Qualitätsentwicklung in der Prävention und Gesundheitsförderung lässt sich nach Ruckstuhl [10] in 3 Phasen unterteilen. In der ersten Phase wurden nach Erfahrungen aus anderen Bereichen die Bedeutung und Potenziale der Qualitätsentwicklung erkannt, wobei die Bewertung von Interventionen im Vordergrund stand. Mit ihrer kriteriengestützten Bewertungshilfe zur Prävention von Übergewicht („Qualitätsraster Adipositas“ 1993) übernahm die BZgA eine Führungsrolle in der Qualitätsentwicklung. Ihre Kampagne zur HIV-Prävention wurde – wie auch viele ihrer anderen Programme – von Beginn an jährlich bezüglich Umsetzung und Wirksamkeit evaluiert [11]. In der zweiten Phase wurden Qualitätskriterien, Instrumente, Modelle und Qualitätssysteme in Anlehnung an Medizin und Management entwickelt (z. B. Qualitätszirkel [12]) und entsprechende Kompetenzen aufgebaut [13]. Die dritte Phase ist gekennzeichnet durch die Entwicklung *präventions- und gesundheitsförderungsspezifischer Qualitätssysteme *und Verfahren von z. T. hoher Komplexität und ihrer ansatzweisen Etablierung; in Infobox 1 werden einige exemplarisch aufgeführt. Hierzu zählen die 12 Good-Practice-Kriterien des 2003 entstandenen Kooperationsverbundes für gesundheitliche Chancengleichheit [14], das Qualitätssicherungsverfahren „QIP – Qualität in der Prävention“ [15, 16], das in der Schweiz entwickelte internetbasierte Qualitäts- und Projektmanagementsystem „quint-essenz“ [17] sowie die partizipative Qualitätsentwicklung [18, 19]. Diese berücksichtigen zentrale Anforderungen an die Prävention und Gesundheitsförderung wie den Einbezug benachteiligter Zielgruppen, deren Erreichbarkeit und Zugangswege.

Es gibt verschiedene Maßnahmen zur Bewertung der Qualität gesundheitsfördernder Einrichtungen und Angebote wie Zertifizierungen und Qualitätssiegel. Es bestehen mehrere *Zertifizierungsverfahren*, z. B. seit 2004 das „Audit Gesunde Schule“ [20] und seit 2006 das „Audit Gesunde KiTa“ ([21]; Infobox 2). Ein *Qualitätssiegel* für gemeinnützige Institutionen, das nicht explizit für Prävention und Gesundheitsförderung entwickelt wurde, ist das „Wirkt-Siegel“ der PHINEO gAG. Qualitätssiegel für Präventionskurse gibt es insbesondere im Gesundheitssport, wo seit dem Jahr 2000 „SPORT PRO GESUNDHEIT“ als Dachmarke fungiert [22]. Weiterentwicklungen erfolgten mit der Einführung des Qualitätssiegels „Pluspunkt Gesundheit – Präventionsgeprüft“ und der Zusammenarbeit mit der Zentralen Prüfstelle Prävention (ZPP, Deutscher Standard Prävention) der gesetzlichen Krankenkassen (siehe unten).

Inzwischen liegen zahlreiche *Instrumente* zur Qualitätssicherung vor. Das Landeszentrum Gesundheit Nordrhein-Westfalen [4] gibt einen Überblick über 11 Instrumente, die sich hinsichtlich der einbezogenen Qualitätsdimensionen (Infobox 3), der Spezifität (zu generischen Systemen siehe Infobox 1), der Art der Beurteilung (intern/extern), des zeitlichen Aufwandes und der anfallenden Kosten unterscheiden. Sie umfassen u. a. Datenbanken mit Instrumenten zur Evaluation, Anleitungen zur Programmplanung, Verfahren zur Definition und Überprüfung von Zielen, Erhebungsinstrumente für spezifische Settings und umfassende Systeme zur Qualitätsentwicklung. Trotz dieser Entwicklung auf vielfältigen Ebenen wird bislang keines dieser Instrumente und Verfahren flächendeckend eingesetzt [23].

## Gesetzliche Regelungen, Empfehlungen und Strukturbildung

Die Entwicklung der Prävention und Gesundheitsförderung sowie ihre Qualitätsentwicklung wurden maßgeblich durch gesetzliche und untergesetzliche Regelungen befördert und unterstützt. Hervorzuheben ist die *Wiedereinführung des § 20 Fünftes Buch Sozialgesetzbuch (SGB V)* im Jahr 2000 mit den erstmals gesetzlich verankerten – und im Präventionsgesetz (PrävG) ausgeweiteten – Anforderungen an Qualität (Bedarf, Zielgruppen, Zugangswege, Inhalt, Methodik, PrävG: intersektorale Zusammenarbeit, wissenschaftliche Evaluation, Erfolgsmessung). Noch im selben Jahr legten die Krankenkassen, wie gefordert, gemeinsam und einheitlich Handlungsfelder und Kriterien vor. Bis heute gilt der *Leitfaden Prävention* [24] als zentrales Element ihrer Qualitätssicherung und -entwicklung. Dieser enthält Umsetzungsanforderungen und Förderkriterien für die Prävention und Gesundheitsförderung in Lebenswelten, für Leistungen der individuellen verhaltensbezogenen Prävention, für die Betriebliche Gesundheitsförderung (BGF) sowie (neu) für die digitale Prävention und Gesundheitsförderung. Hierzu zählen neben definierten Qualifikationen der Anbieter auch der Einbezug der Zielgruppen und Stakeholder während des gesamten Prozesses. Neben der Struktur- und Prozessqualität wird stichprobenartig die Ergebnisqualität erfasst. Einen ersten Ansatz zur Erfassung der Wirksamkeit im Individualansatz legten Kliche et al. [25] vor.

Die gesetzlich geforderte Prüfung der Qualität der Präventionskurse nach einem einheitlichen Verfahren und ihrer Zertifizierung erfolgt durch die 2014 eingerichtete Zentrale Prüfstelle Prävention (ZPP, www.zentrale-pruefstelle-praevention.de) durch 4 handlungsfeldbezogene Prüfteams. Seitdem erhielten ca. 250.000 Präventionskurse das Qualitätssiegel „Deutscher Standard Prävention“.

Neben dem Leitfaden Prävention der Gesetzlichen Krankenversicherung (GKV; [24]) liegen inzwischen auch für alle anderen Sozialversicherungen Qualitätskriterien für die Prävention und Gesundheitsförderung vor, wenn auch größtenteils weniger ausdifferenziert: der Leitfaden „Prävention in stationären Pflegeeinrichtungen“ des Spitzenverbands Bund der Krankenkassen (GKV-Spitzenverband; [24]), Qualitätskriterien im Präventionsfeld Gesundheit im Betrieb der Gesetzlichen Unfallversicherung [26] sowie das „Rahmenkonzept zur Umsetzung der medizinischen Leistungen zur Prävention und Gesundheitsförderung“ der Deutschen Rentenversicherung [27].

Einen Schub für die Qualitätsentwicklung gab das 2015 in Kraft getretene *Präventionsgesetz (PrävG)* mit der Stärkung der Prävention und Gesundheitsförderung in Lebenswelten und der Betrieblichen Gesundheitsförderung, dem Einbezug weiterer Akteure und der Forderung nach abgestimmten Strukturen. Die in diesem Kontext entwickelten *Bundesrahmenempfehlungen* (Erstfassung 2016, überarbeitet 2018 [28]) mit ihrer Orientierung am Public Health Action Cycle zielen ab auf die „Sicherung und Weiterentwicklung der Qualität“ und die „Zusammenarbeit der … zuständigen Träger und Stellen“ (§ 20d Abs. 3 SGB V). Zur Umsetzung der nationalen Präventionsstrategie liegen seit Juli 2018 die *Landesrahmenvereinbarungen* (§ 20f SGB V) für alle Bundesländer vor, die auch der Stärkung der kommunalen Steuerungsfunktion in der Prävention und Gesundheitsförderung dienen. In fast allen Ländern wurden neue dialog-, abstimmungs- oder entscheidungsorientierte Strukturen geschaffen und die Zieleplanung neu belebt. Die Schlüsselrolle der Kommunen, der gesetzlich geforderte Einbezug des Öffentlichen Gesundheitsdienstes (ÖGD) sowie der Kinder- und Jugendhilfe sind allerdings nur eingeschränkt in den Landesrahmenvereinbarungen verankert [29], was als deutliches Qualitätsproblem wahrgenommen wird [30].

Wesentlich für die Weiterentwicklung der Prävention und Gesundheitsförderung ist der *Präventionsbericht *(§ 20d SGB V), der 2019 erstmals erschien [31]. Dieser gibt u. a. einen Überblick über die Umsetzung des Präventionsgesetzes, einschließlich der geforderten Qualitätsentwicklung. Der gesetzlich geforderte Einbezug der Gesundheitsberichterstattung zumindest auf Bundesebene wird als Meilenstein gesehen [32]. Er führte zu einer Auseinandersetzung bezüglich Methodik, geeigneter Indikatoren, Akteursbeteiligungen, Wirkungsmodellen, Anschlussfähigkeit, aber auch Verantwortlichkeiten einer guten Präventionsberichterstattung [33]. Auch wenn es keine dezidierten Vorgaben für die Länder gibt, haben diese begonnen, sich auf einen gemeinsamen Kernindikatorensatz zu verständigen. Bereits im Vorfeld des Präventionsgesetzes wurden in einigen Ländern kommunale Netzwerke für eine Präventionsberichterstattung aufgebaut, die in den letzten Jahren zu einer vermehrten Nutzung von Gesundheitsberichten als Diskussions- und Planungsgrundlage führten [34].

Wie eine Bestandsaufnahme im Vorfeld des Präventionsgesetzes [2] zeigt, bestehen neben Vorgaben zur Qualitätssicherung im Sozialversicherungsrecht zahlreiche, auch für die Prävention und Gesundheitsförderung anschlussfähige rechtliche Regelungen, Verwaltungsvorschriften und Empfehlungen für die einzelnen Einrichtungen bzw. Settings. Diese variieren allerdings erheblich zwischen den Ländern bezüglich ihrer Implementation, der Ausdifferenzierung von Routinen und der Vorgaben zur Akzeptanz von Instrumenten zur Qualitätsentwicklung. Etwa die Hälfte der Gesetze des ÖGD enthält Angaben zur Qualitätsentwicklung, ein expliziter Bezug zur Prävention und Gesundheitsförderung ist jedoch sehr selten [2, 35].

Mit dem *Bundeskinderschutzgesetz (BKiSchG) *wurden 2012 bundesweit *Frühe Hilfen* mit ihren niederschwelligen intersektoralen Angeboten für Schwangere und Familien mit Säuglingen und Kleinkindern in der Kommune gesetzlich verankert. Interprofessionelle, hilfesystemübergreifende Qualitätszirkel unterstützen die Vernetzung sowie den fachlichen und fallbezogenen Austausch. Die Förderung der Qualitätsentwicklung in den Kommunen erfolgt in einem mehrjährigen dialogisch und partizipativ gestalteten Prozess. Grundlage bildet der *Qualitätsrahmen Frühe Hilfen*, der 9 Qualitätsdimensionen umfasst (Grundidee, Zielbestimmung, Netzwerk, Planung, politisch-strukturelle Verankerung vor Ort, Qualifizierung und interprofessionelles Lernen, Zusammenarbeit mit der Familie, Qualität von Angeboten, Dokumentation und Evaluation; [36]).

## Forschung zur Qualitätsentwicklung

Forschung zur Qualitätsentwicklung erfolgt meistens im Rahmen übergreifender Fragestellungen und weniger in expliziten Studien zu dieser Thematik. Die seit 2004 vom Bundesministerium für Bildung und Forschung geförderte Forschung zur Prävention und Gesundheitsförderung ermöglicht hierzu Projekte (z. B. [10, 21, 37–40]), ebenso unterstützt insbesondere die BZgA entsprechende Entwicklungen und Untersuchungen (z. B. [9, 41]). Schwerpunkte bilden die Entwicklung und Erprobung von Instrumenten, z. B. zur Kapazitätsentwicklung in Kommunen [37] und zur einfachen onlinegestützten Selbstevaluation von Interventionen [41], sowie Erhebungen zu Rahmenbedingungen und zur Umsetzung (z. B. [2, 9, 23, 42]).

Studien zur *Wirksamkeit* der Qualitätssicherung sind insgesamt rar. Auswirkungen einer Zertifizierung zeigen Untersuchungen zu Kitas und Schulen. Das „Audit Gesunde KiTa“ [20] und das „Audit Gesunde Schule“ [21] werden von den Teilnehmenden als sehr unterstützend erlebt und führen zu dauerhaften Struktur- und Prozessverbesserungen. Ebenso trägt das Zertifikat „Bewegte Kita“ zu einer bewegungsfreundlicheren Gestaltung und erhöhten Bewegungsaktivität der Kinder bei [38]. Eine Untersuchung des Gesamtzertifikats „Gesundheitsfördernde Schulen“ zeigt (außer bei dem Teilzertifikat Bewegung) keinen positiven Zusammenhang mit dem gesundheitsrelevanten Verhalten und den Einstellungen der Jugendlichen [39]. Kritisch anzumerken sind eine teilweise sehr geringe Teilnahmequote [38, 39], ausschließlich Befragung von Expertinnen und Experten [20, 21] und die Nichterfassung der Veränderungen im Setting [39].

Aktuell wird im Rahmen einer kontrollierten Studie mit über 40 Kommunen die Effektivität des international verbreiteten, in den USA evaluierten und nach Deutschland transferierten Präventionssystems „Communities That Care (CTC)“ untersucht. Hierzu werden strukturierte Verfahren der Qualitätsentwicklung eingesetzt sowie die Prozess- und Ergebnisqualität evaluiert. Die Studie enthält auch eine ökonomische Evaluation [40].

## Umsetzung und Handlungsbedarf

Ein Indikator für die Relevanz von Qualitätsentwicklung für Einrichtungen und Akteure ist ihre *organisationale Verankerung*. Diese hat sich in dem letzten Jahrzehnt verändert. Während 2009/2010 Qualitätssicherung und -entwicklung noch überwiegend situativ, improvisiert und vom Leitungspersonal miterledigt wurde [43], verfügen inzwischen die meisten Organisationen über spezialisierte Beauftragte [44]. Dieses wird von dem Präventionsbericht [31] weitgehend bestätigt. Danach sind in der Regel (zu 70–80 %) Mitarbeitende neben anderen Aufgaben für Qualitätssicherung zuständig, eher selten (zu 10–20 %) jedoch ausschließlich. Anders sieht es bei Kommunen und Einrichtungen der organisierten Zivilgesellschaft aus, die zu 30 % bzw. 20 % keine Zuständigkeit angeben. Nach dem Präventionsbericht weisen viele Akteure Ausgaben für Qualitätsentwicklung und Evaluation nicht separat aus, oft liegt keine explizite *Finanzierung* vor. Die Teilnahme an einer Zertifizierung wurde bei Kitas über die Träger (35 %), das eigene Budget (29 %), Sponsoren (26 %) und Spenden (10 %) finanziert [20]. Anders sieht es bei Schulen aus. Finanzierungsquellen waren hier insbesondere vollständig oder anteilig die Träger (allein: 28 %, anteilig: 13 %) und Krankenkassen [21].

Die breite Palette der *Instrumente zur Qualitätsentwicklung* wird von den meisten Akteursgruppen zu über einem Drittel (sehr) oft eingesetzt [31]. Eine Ausnahme bilden solche Kommunen, in denen erheblicher Entwicklungsbedarf gesehen wird. Deutliche Unterschiede in der Nutzung von Instrumenten zwischen den Settings ermittelte eine Bestandsaufnahme zur Qualitätssicherung im Vorfeld des Präventionsgesetzes [23]. Während in den strukturierten Einrichtungen Kita und Schule eher Qualitätsmanagementkonzepte zum Einsatz kommen, die in Prozesse der Organisationsentwicklung einfließen, nutzen Quartiere vor allem niedrigschwellige, offene Verfahren wie Planungskonferenzen, Stadtteilbegehungen, Stufen der Partizipation oder den Good-Practice-Ansatz. In allen Settings wurden eher generische Instrumente zur Qualitätssicherung eingesetzt und seltener solche, die speziell im Bereich Prävention und Gesundheitsförderung entwickelt wurden [23].

Dies ist auch trägerspezifisch vorgegebenen Qualitätsmanagementsystemen geschuldet [2]. So zeigt eine Analyse der *Rahmenhandbücher bzw. Qualitätsmanagementsysteme* von Wohlfahrtsverbänden sowie der einrichtungsbezogenen Materialen zur Qualitätsentwicklung ihrer Kitas, dass gesundheitsbezogene Ziele und Prozessbeschreibungen nur selten konkret formuliert werden [42]. Da die übergreifenden Vorgaben handlungsrelevant sind, würde eine entsprechende Anpassung eine qualitätsgesicherte Gesundheitsförderung erleichtern und Zielkonflikte vermeiden. Eine flächendeckende Umsetzung kann allerdings nur durch eine begleitende *Qualifizierung* aller Beteiligten erreicht werden [42]. Wie wichtig entsprechende Kompetenzen sind, zeigt eine Befragung von 595 Akteuren in Bayern (Rücklauf 23 %), die grundlegende Aspekte der Planungsqualität nicht hinreichend beachten. So berücksichtigt knapp die Hälfte die vorliegende Evidenz nicht, ein Viertel führt keine Bedarfsanalysen durch [45].

Von der Bundesinitiative *Frühe Hilfen* wird Qualitätsentwicklung in 573 Kommunen gefördert. Nach einer Erhebung zur Umsetzung (Teilnahme: 67 %) nutzen 68 % formale und 19 % informelle Verfahren [46]. In 12 % der Kommunen ist Qualitätsentwicklung kein regelmäßiges Thema. Die eigens entwickelten Unterstützungsmaterialien [36] sind zwar 3 Vierteln der Kommunen bekannt, werden allerdings nur von der Hälfte der Kommunen eingesetzt. Positiv ist hervorzuheben, dass eine Auseinandersetzung mit Qualitätsentwicklung besonders häufig in der systemübergreifenden Zusammenarbeit erfolgt (89 %).

Auch wenn Qualitätssicherung und -entwicklung insgesamt als wichtig für die Selbstreflektion gesehen werden, stellen im Alltag begrenzte finanzielle Mittel, fehlendes qualifiziertes Personal sowie die hohen Zeitanforderungen zentrale *Hemmnisse* in der Umsetzung dar [20, 21, 23, 46]. Zudem bestehen Befürchtungen, dass die Ergebnisse zur fiskalischen Steuerung genutzt werden [16]. Von den Akteuren wird ein noch nicht aufgelöstes Dilemma gesehen: einerseits der Wunsch nach der Standardisierung von Maßnahmen, um die Transparenz und Verlässlichkeit, aber auch die politische Legitimation zu erhöhen, und andererseits die Notwendigkeit, Qualitätssicherung mit niedrigschwelligen Instrumenten flexibel an die spezifischen Voraussetzungen anzupassen [16, 42, 47]. Bezüglich des Verfahrens QIP, das durchaus für einen breiten Einsatz geeignet ist, sehen Kliche et al. [16] für die nicht erreichte Marktakzeptanz auch Gründe auf Anbieterseite, die bei zu geringer Nutzung nicht gewährleistet werden kann: eine aufwendige Systempflege einschließlich der erforderlichen kontinuierlichen Sichtung und Einarbeitung der neuen Evidenz, und Erhalt eines geschulten Reviewerpools.

*Handlungsbedarf *besteht damit in der Finanzierung und dauerhaften strukturellen Verankerung von Qualitätsentwicklung, in der Qualifizierung, in der noch immer als unzureichend erlebten Vernetzung sowie in der Weiterentwicklung der Gesetzgebung [31, 44, 47]. Angestrebt werden sollte eine Harmonisierung und/oder Anpassung der einzelnen Ländergesetze (z. B. des ÖGD) sowie der Bildungs- und Orientierungspläne etc., um förderliche Rahmenbedingungen zur Umsetzung der Prävention und Gesundheitsförderung und zu ihrer Qualitätsentwicklung zu schaffen. Nicht zuletzt sollte auch in der Prävention und Gesundheitsförderung eine partiell bereits angestoßene Fehlerkultur (weiter) gefördert werden, die es ermöglicht, nicht nur aus Best-Practice-Projekten, sondern auch aus misslungenen Projekten zu lernen [1]. Dies setzt eine Offenheit von Akteuren und Forschenden sowie den einschlägigen Publikationsorganen voraus, entsprechende Erfahrungen zu veröffentlichen.

Auszubauen ist eine kleinräumige *Präventionsberichterstattung*, die sich an den Qualitätsdimensionen orientiert und, neben der Gesundheitsberichterstattung zur Bedarfsanalyse, eine Interventionsberichterstattung zur Planung und Umsetzung sowie eine Ergebnisberichterstattung enthält [30]. Dabei sollten auch die in den vergangenen Jahren veränderten Konzepte mit der vermehrten Ressourcenorientierung, Verhältnisprävention und dem Health-in-All-Policies-Ansatz abgebildet werden [33]. Besonderer Beachtung bedürfen dabei auch ethische Reflexionen, die auf unterschiedlichen Ebenen der Berichterstattung z. B. Wertungen beinhalten, Diskriminierungen vornehmen, das Präventionsdilemma verstärken und Nebenwirkungen vernachlässigen [48].

Noweski et al. [47] konstatieren 2 relativ unverbundene Qualitätsdiskurse in der Prävention und Gesundheitsförderung: die spezifische und unspezifische Qualität von Prävention und Gesundheitsförderung. Während Erstere Forschungen zur Wirksamkeit einzelner Maßnahmen umfasst und in Leitlinien eingeht, wird die zweite von normativen, gesundheitspolitischen und formalen Setzungen geprägt. Das Präventionsgesetz (PrävG) besitzt das Potenzial, ein einheitlicheres Vorgehen in der Qualitätsentwicklung zu stärken und Standards zu entwickeln. Wesentlich zur Förderung der Qualitätsentwicklung in den nächsten Jahren können beitragen: ein settingbezogener und -übergreifender Austausch über Qualität, eine bundesweite Errichtung von Qualitätsnetzwerken sowie der Ausbau einer Koordinierungs- und Transferstruktur für Qualitätsentwicklung in der Prävention und Gesundheitsförderung in Lebenswelten [11].

## Fazit

Die Qualitätsentwicklung in der Prävention und Gesundheitsförderung in den letzten 30 Jahren ist eng mit der allgemeinen Entwicklung der Prävention und Gesundheitsförderung verbunden – mit der ihr gegenüber verstärkten Akzeptanz und der vermehrten theoretisch-konzeptionellen sowie empirischen Fundierung, mit ihrer rechtlichen und curricularen Verankerung sowie ihrer Strukturierung und Ausdifferenzierung. Dabei sind eine deutliche Auseinandersetzung mit Qualitätssicherung sowie deren Weiterentwicklung zu verzeichnen.

Inzwischen liegt eine größer werdende Palette an Instrumenten zur Qualitätssicherung und -entwicklung vor, die explizit für die Prävention und Gesundheitsförderung konzipiert worden sind – z. T. spezifisch für Handlungsfelder, Settings bzw. Einrichtungen, z. T. zum übergreifenden Einsatz. Ebenso liegen komplexe Qualitätsmanagementsysteme vor. Daneben existieren generische Verfahren, die von den Trägern und deren Einrichtungen eingesetzt werden, bisher weitgehend ohne Bezug zur Prävention und Gesundheitsförderung. Hier bedarf es zukünftig einer Verzahnung. Bislang kann von einer flächendeckenden und kontinuierlichen Verankerung und Umsetzung von Qualitätssicherung in der Prävention und Gesundheitsförderung noch längst nicht gesprochen werden.

Qualitätssicherung und -management differieren erheblich zwischen der Individualprävention und den Settings bzw. Lebenswelten, abhängig auch von der Entwicklung der Prävention und Gesundheitsförderung sowie den jeweiligen (gesetzlichen) Anforderungen. Ein erheblicher Nachholbedarf besteht in der kommunalen Gesundheitsförderung, die erst seit Kurzem eine verstärkte Aufmerksamkeit und Förderung erfährt. Sie zeichnet sich als Dachsetting zugleich durch besonders komplexe Strukturen und die Integration unterschiedlicher Bereiche mit eigenem Qualitätsverständnis aus (Gesundheit, Bildung, soziale Arbeit). Systematische und vergleichbare Überblicke zur Qualitätssicherung in den einzelnen Handlungsfeldern und Settings liegen bislang nicht vor.

Belege zu erreichten Verbesserungen durch Qualitätssicherung und -management im Praxisalltag sind äußerst rar. Vielmehr liegen wissenschaftliche Evaluationen zur Wirksamkeit von Interventionen vor. Diese verdeutlichen die hohe Relevanz der Konzeptqualität und liefern Hinweise zur Prozessoptimierung. Forschungsbedarf besteht zur Wirksamkeit von Qualitätssicherung und -management in der Prävention und Gesundheitsförderung sowie zu dem Verhältnis von Aufwand und Nutzen.

Eine flächendeckende, kontinuierliche Qualitätssicherung und -entwicklung in der Prävention und Gesundheitsförderung erfordert eine strukturelle Verankerung bei hinreichender Budgetierung, Qualifizierung und Monitoring. Neben der bereichsspezifischen Weiterentwicklung sollte ein übergreifender Diskurs und Konsens angestrebt werden. Das Präventionsgesetz hat bereits im Vorfeld Impulse gesetzt und bietet Potenzial zur Sicherung und Weiterentwicklung der Qualität in der Prävention und Gesundheitsförderung.

### Infobox 1 Beispiele für Instrumente und Systeme zur Qualitätsentwicklung in Prävention und Gesundheitsförderung

*Good Practice* beinhaltet 2 zentrale Elemente: Die Good-Practice-Kriterien und die Datenbank mit Projekten der soziallagenbezogenen Gesundheitsförderung. Kommunalen Gesundheitsfachkräften helfen 12 Good-Practice-Kriterien bei der Reflexion von Stärken und Schwächen des eigenen Projektes. Um die Qualität besonders niederschwelliger Interventionen zu verbessern, werden Maßnahmen, die von extern als gut bewertet wurden, in einer Datenbank als Good Practice aufgenommen und zur Nachahmung bzw. Anpassung an andere Kontexte empfohlen. Die Datenbank (www.gesundheitliche-chancengleichheit.de) umfasst inzwischen über 3100 Projekte (Stand: 01.12.2021). Der Schwerpunkt liegt auf der Planungs- und Prozessqualität [14]. Good Practice wurde im Rahmen des Kooperationsverbundes für gesundheitliche Chancengleichheit entwickelt. Dieser wurde von der Bundeszentrale für gesundheitliche Aufklärung initiiert und umfasst inzwischen 75 Kooperationspartner.

*QIP – Qualität in der Prävention* wurde evidenzbasiert und expertengestützt entwickelt. Grundlage bilden 7 Haupt- und 22 Teilqualitätsdimensionen zur Konzept‑, Struktur‑, Prozess- und Ergebnisqualität. QIP umfasst (1) die Dokumentation zentraler Qualitätsmerkmale in den 4 Qualitätsebenen, (2) die Begutachtung und Bewertung dieser Informationen im Peer-Review-Verfahren durch externe geschulte Fachkräfte, (3) die Sammlung der Daten in einer Referenzdatenbank für wissenschaftliche Analysen und Benchmarks sowie (4) die Rückmeldung der Ergebnisse an die beteiligten Institutionen und die Politik. QIP wurde vom Universitätsklinikum Hamburg-Eppendorf in Kooperation mit der Bundeszentrale für gesundheitliche Aufklärung entwickelt [15, 16].

*quint-essenz* ist ein Qualitätssystem, das Qualitätsentwicklung und Projektmanagement miteinander verknüpft und Aspekte des Total Quality Management (TQM) berücksichtigt. Kernstück sind 24 Kriterien, die 6 Bereichen zugeordnet sind und den gesamten Projektablauf von der Konzeption bis zur Evaluation umfassen. Sie bieten eine Vielfalt an Instrumenten, Informationen und praktischen Tipps. Das System kann zur Verbesserung aller Qualitätsdimensionen im gesamten Projektablauf oder auch punktuell eingesetzt werden [17]. quint-essenz wurde von der privatrechtlichen Stiftung Gesundheitsförderung Schweiz als nationaler Referenzrahmen für Präventions- und Gesundheitsförderungsprojekte entwickelt. Die von 2001 bis 2020 bestehende Website zu quint-essenz wurde 2021 in die neue Plattform „Good-Practice“ überführt (www.good-practice.ch). Im Rahmen der bundesweiten Initiative „IN FORM“ erfolgte von 2008 bis 2011 eine Anpassung für Deutschland.

### Infobox 2 Zertifizierung und Qualitätssiegel zur Bewertung von gesundheitsfördernden Einrichtungen

**Zertifizierung**

*Audit Gesunde KiTa* ist ein Zertifizierungsverfahren, das 8 Qualitätsfelder beinhaltet, u. a. zu Arbeitsbedingungen, zum gesundheitsförderlichen Zustand der KiTa, zur Gesundheit der Kinder und des Personals sowie zu Partnerschaften und Qualitätsmanagement. Das seit 2006 angebotene Verfahren wird in 7 Bundesländern in bislang 295 Kitas eingesetzt (Stand: 01.12.2021, www.lvg-lsa.de/beratungsstellen/audit-gesunde-kita).

*Audit Gesunde Schule* umfasst 55 Kriterien in 5 Kategorien zu Schulbedingungen, -klima, Unterricht, Qualitätsmanagement und Gesundheitskompetenz. Mit dem seit 2004 angebotenen Audit Gesunde Schule wurden in 9 Bundesländern bislang 325 Schulen evaluiert (Stand: 01.12.2021, www.lvg-lsa.de/beratungsstellen/audit-gesunde-schule/).

Beide Audits wurden von der Landesvereinigung für Gesundheit Sachsen-Anhalt gemeinsam mit Praktikerinnen und Praktikern entwickelt und erprobt. Sie umfassen eine Selbst- und Fremdbewertung mit Entwicklungszielen bis zur Rezertifizierung nach 3 Jahren.

**Qualitätssiegel für gemeinnützige Einrichtungen**

*Wirkt-Siegel:* Das Analyse- und Beratungshaus PHINEO gAG möchte die Qualität in Non-Profit-Organisationen und gemeinnützigen Institutionen fördern und die Wirkungen gemeinnütziger Arbeit abbilden. Anders als die anderen genannten Systeme wurde es nicht explizit für die Prävention und Gesundheitsförderung entwickelt. Der Fokus von PHINEO liegt auf der Ergebnisqualität (Wirkungsorientierung). Handbücher und Arbeitshilfen, Workshops, Analysen und Beratung zur Förderung der Wirksamkeit unterstützen die Planung, Durchführung und Evaluation von Maßnahmen. Die Begutachtung erfolgt durch eine Expertenkommission und entscheidet über die Vergabe des Wirkt-Siegels. PHINEO entstand 2007 aus einem Projekt der Bertelsmann Stiftung (www.phineo.org).

### Infobox 3 Qualitätsdimensionen in der Prävention und Gesundheitsförderung – Entwicklungen und Beispiele für Instrumente

Neben den etablierten Dimensionen der Struktur‑, Prozess- und Ergebnisqualität ist für die Prävention und Gesundheitsförderung die Planungsdimension (auch Konzept- bzw. Assessmentdimension genannt) relevant, die auch ein zentrales Element des Public Health Action Cycle ist.

*Planungsqualität* bezieht sich auf die grundlegende Konzeption und ihre theoretisch-empirische Fundierung, die wesentlich über den Erfolg oder das Scheitern eines Vorhabens entscheidet. Zentrale Elemente sind u. a. die Ermittlung des Bedarfs und der Bedürfnisse, die Definition der Ziele und Zielgruppen, die Evidenzbasierung der Intervention und des Vorgehens, eine Kontextanalyse bezüglich bestehender Ansätze und ihrer Übertragbarkeit, Strategien im Umfeld sowie einzubeziehende Kooperationspartner [1]. Für die einzelnen Aspekte wurden zahlreiche Unterstützungshilfen entwickelt. Inwieweit sich diese Instrumente für einen breiten Einsatz eignen und flächendeckend empfohlen werden können, muss sich in den nächsten Jahren zeigen.

Die in den vergangenen Jahren eher unsystematisch und kaum geförderte Aufbereitung der Evidenz zur Prävention und Gesundheitsförderung wurde im Zuge des Präventionsgesetzes zu Lebenswelten verstärkt. Inzwischen liegen zahlreiche Berichte zu verschiedenen Themen mit unterschiedlicher Evidenz vor [49]. Einen Überblick über die Evidenz von 100 Programmen (Stand 31.10.2021) gibt die Datenbank *Grüne Liste Prävention* (www.gruene-liste-praevention.de), die im Jahr 2011 online gestellt wurde. Sie enthält bislang vor allem Interventionen zur Förderung der psychischen Gesundheit und zur Prävention von Risikoverhaltensweisen wie Sucht, Gewalt und sozial unerwünschtem Verhalten.

*Strukturqualität* mit ihrem Fokus auf Rahmenbedingungen wie Qualifizierung, Personal und Teamkontinuität, Ausstattung und Finanzierung ist ein zentraler Bestandteil im Leitfaden Prävention [24]. In der Qualitätsentwicklung lagen in den vergangenen Jahren die Schwerpunkte auf der Konzeption und Erprobung von Qualifizierungsmodulen [6], den Schulungen verschiedener Einrichtungen (z. B. Landesvereinigungen für Gesundheit, Kooperationsverbund für gesundheitliche Chancengleichheit) sowie der Vernetzung von Schlüsselpersonen [1].

Auch die *Prozess- und Ergebnisqualität* erfuhren eine Ausdifferenzierung. Vermehrt beachtet wurde in den vergangenen Jahren die kommunale Prävention und Gesundheitsförderung, für die inzwischen für alle Qualitätsdimensionen entsprechende Handlungsempfehlungen, Instrumente, Checklisten etc. vorliegen (z. B. [5, 37, 48, 50]).
